# Photomechanical Crystals as Light-Activated Organic Soft Microrobots

**DOI:** 10.1021/jacs.4c08320

**Published:** 2024-10-11

**Authors:** Ibrahim Tahir, Ejaz Ahmed, Durga Prasad Karothu, Filmon Fsehaye, Jad Mahmoud Halabi, Panče Naumov

**Affiliations:** † Center for Smart Engineering Materials, New York University Abu Dhabi, PO Box 129188, Abu Dhabi 129188, United Arab Emirates; ‡ Smart Materials Lab, New York University Abu Dhabi, PO Box 129188, Abu Dhabi 129188, United Arab Emirates; § Research Center for Environment and Materials, Macedonian Academy of Sciences and Arts, Bul. Krste Misirkov 2, Skopje 1000, Macedonia; ∥ Molecular Design Institute, Department of Chemistry, New York University, 100 Washington Square East, New York, New York 10003, United States

## Abstract

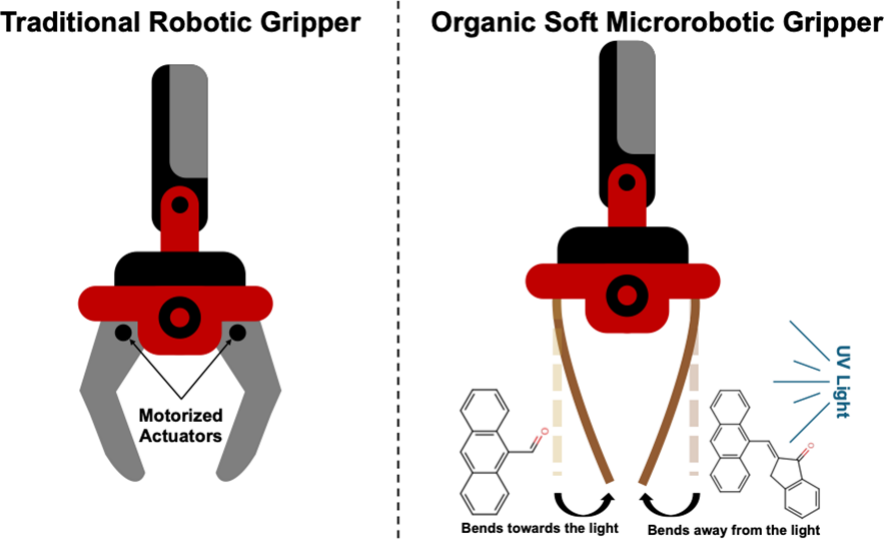

In the field of materials science, dynamic molecular crystals have attracted significant attention as a novel class of energy-transducing materials. However, their development into becoming fully functional actuators remains somewhat limited. This study focuses on one family of dynamic crystalline materials and delves into exploring the efficiency of conversion of light energy to mechanical work. A simple setup is designed to determine a set of performance indices of anthracene-based crystals as an exemplary class of dynamic molecular crystals. The ability of these crystals to reversibly bend due to dimerization is realistically assessed from the perspective of the envisaged soft robotics applications, where wireless photomechanical grippers manipulate and assemble microscopic objects driven and controlled by light instead of lines and motors. The approach described here not only guides the quantification of responsive molecular crystals’ actuation potential but also aims to attract an interdisciplinary interest to further develop this class of materials into controllable all-organic actuating elements to be used in microrobotics for engineering or biomedicine.

## 1 Introduction

Transformation of the energy of external stimuli into mechanical motion that can perform work by utilizing stimuli-responsive materials represents a subject of profound scientific and technological interest. Such responsive materials can present dramatic mechanical motions under the action of external effectors such as irradiation with ultraviolet (UV), visible or infrared light,^1^ heating,^2^ application of external force,^3^ action of solvent,^4^ electrical fields,^5^ and others.^6−10^ Dynamic molecular crystals have recently emerged as a promising class of energy-transducing materials^8,9^ with the potential to revolutionize several technological fields, including sensing, bioimaging, data storage, deformation detection, property switching, and security-related systems. Among these stimuli, light is currently attracting an increasing interest due to its advantages, including non-contact control, availability from natural and non-natural sources, cost-effectiveness, and the opportunity to manipulate matter.^7^ Light-driven actuation of mechanically responsive molecular crystals is relevant to understanding the physicochemical foundations of the light-matter interaction and has also been considered for sensing, actuation, and soft robotics.^11−14^ Deformations or motions of photoreactive crystals have been demonstrated with diarylethenes,^15−17^ anthracenes,^18−21^ azobenzenes,^22−27^ salicylideneanilines,^28,29^ and other compounds,^30−38^ and the underlying photoinduced processes can be photodimerization,^18−21^ photoisomerization,^22−27^ or photopolymerization.^34^ While various materials, including azobenzenes,^39,40^ furylfulgides,^41^ dibenzobarralenes,^42^ diarylethenes,^15,43^ salicylideneanilines,^44^ hydrazones,^37,45^ styrylbenzoxazoles,^46^ and styrylbenzothiazoles,^46^ have been explored for their photomechanical responses, each presents challenges such as complex synthesis,^15,43,46^ high temperature required for unbending,^45^ limited bending curvature,^44^ or irreversible photomechanical behavior.^37^ These challenges highlight the advantages of anthracene-based derivatives, which offer ease of preparation, suitable crystal dimensions, significant bending curvature, and most importantly, quick and reversible photomechanical responses under different conditions, such as gentle heating, opposite-face irradiation, or recovery in ambient conditions. These characteristics make anthracene-based crystals particularly well-suited for applications like microgripping, where precision and reversibility are crucial. Many of the reports on light-responsive crystals, however, remain phenomenological; they are limited to the observations of these phenomena and lack deeper insight into the mechanical forces that develop during the underlying processes. The research presented here focuses on establishing an “energy budget” for light-to-work energy conversion by photoinduced bending in molecular crystals. By measuring the relevant parameters and employing specific performance indices for the photobending of four crystalline anthracene-based crystals—materials whose photomechanical properties have been studied extensively by Al-Kaysi, Bardeen, and the collaborators,^1,9,18−20,47−49^—we aim to comprehensively characterize these materials on the same scale as the commonly used actuators. We find that the bending molecular crystals demonstrate an operating range comparable to microactuators, such as microelectromechanical systems (MEMS), while displaying a remarkable work-generating capacity and dynamic performance. Their performance is suited for micromotor drivers in mechanical positioning and microgripping tasks. By using mechanical characterization and numerical simulations, we also expedite the integration of the dynamic molecular crystals into soft microrobotics applications, where their unique capabilities can be utilized for useful purposes. In microrobotics, the ability to manipulate crystals that move in opposite directions upon exposure to light is crucial for tasks such as gripping and object manipulation. Therefore, we selected both anthracene-based derivatives that bend away from the light source and those that bend toward it. This work contributes to advancing stimuli-responsive materials and their potential transformative impact on emerging technologies.

## 2 Results and Discussion

### 2.1 Preparation and Bending

**Figure 1:**
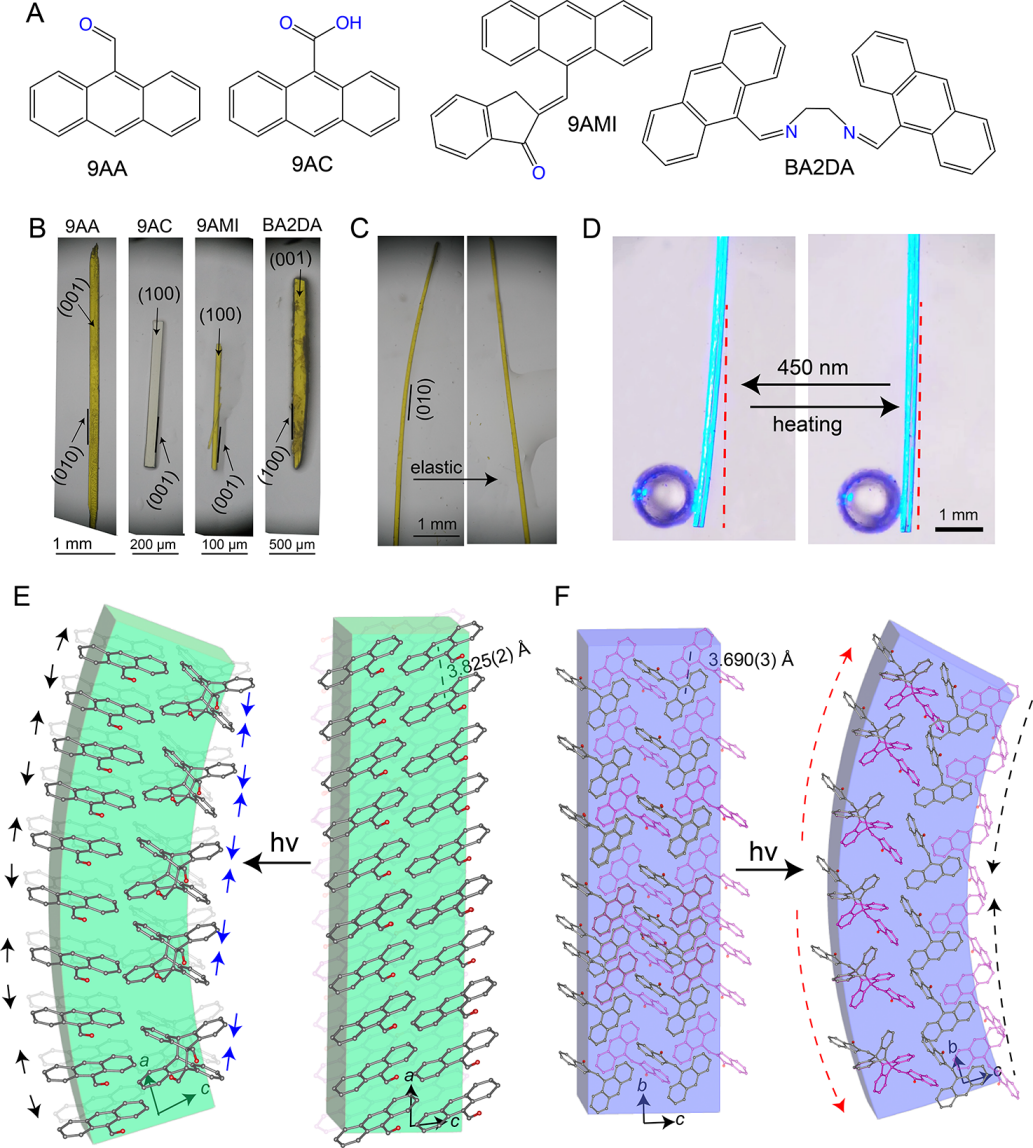
Structure and photomechanical actuation of the anthracene-based dynamic crystals. (A,B) Molecular structures (A) and optical images of crystals (B) of 9-anthranaldehyde (9AA),^48^ 9-anthracenecarboxylic acid (9AC),^18^ (*E*)-2-(9-anthrylmethylene)-1-indanone (9AMI),^49^ and 1,2-*bis*[(anthracen-9-ylmethylene)amino]ethane (BA2DA).^9^ (C) Crystals of 9AA showing elastic response to mechanical stimulus. (D) Photobending of a plank of 9AA upon exposure to UV irradiation. (E) Molecular packing in the crystal of 9AA viewed along the crystallographic *b*-axis showing dimer formation and schematic representation of the mechanism responsible for the UV-inducted bending toward the light. (F) Head-to-tail packing in the crystals of 9AMI viewed along the crystallographic *a*-axis and schematic illustration of the dimerization that results in bending away from the light.

Four anthracene-based derivatives known from the literature, namely, 9-anthranaldehyde (9AA),^48^ 9-anthracenecarboxylic acid (9AC),^18^ (*E*)-2-(9-anthrylmethylene)-1-indanone (9AMI),^49^ and 1,2-*bis*[(anthracen-9-ylmethylene)amino]ethane (BA2DA),^9^ (Figure 1A), were synthesized and/or recrystallized to obtain slender crystals (Figure 1B,C). Based on the structural analysis of single crystals of 9AA, it is known that the molecular arrangement is represented by a J-type aggregation pattern.^48^ The molecular packing viewed on the major planes is shown in Figure 1E. In all four molecules, the molecules are stacked along the crystal growth direction which is along the length of the crystals. The distance between adjacent molecules in all four crystals is shorter than the distance required for photodimerization. When these crystals are illuminated with UV light, the adjacent molecules undergo photodimerization (Figure 1D) and afford photodimers by formation of new bonds (Figure 1E,F).

Five single crystals of each compound were tested under similar conditions, by fixing one of their ends to a glass capillary to form a cantilever. Upon irradiation of one of their facets with UV (365 nm) or blue LED light (470 nm), the crystals bent reversibly due to photodimerization occurring at their surface (Figure 1D and SI Figure S1). The bending is due to compressive or tensile surface strains generated by the molecular transformation that cause the crystal to bend toward or away from the light, respectively, as the dimerization reaction continues to propagate into the crystal. However, the bending directions varied across the compounds: it was observed that crystals of 9AA bent toward the light, while 9AC, 9AMI, and BA2DA bent away from it. This opposite bending behavior is particularly advantageous for robotic applications where coordinated movements in opposite directions are required, such as in the operation of soft microrobots designed for gripping or manipulating objects.^50,51^ While other materials like azobenzene derivatives have shown similar photomechanical responses, the anthracene-based derivatives used here offer the unique advantage of having different materials that bend in the opposite directions while being irradiated with the same light source. Since the photodimerization is reversible, once the source of irradiation was switched off, the crystals recovered their original straight shape slowly in visible light, due to the decomposition of the dimers to monomers. The recovery of the straight shape can be accelerated by gentle heating up to 60 °C, allowing the crystal to revert to its original form within 1 min, or by irradiation from the opposite direction, achieving full unbending in approximately 5 min. However, if the crystal is left at room temperature in the absence of any irradiation, including ambient light (i.e., in the darkness), the unbending is partial. These processes of photodimerization and subsequent bending occur on a relatively short time scale, typically within seconds to minutes, and their reversibility is crucial for potential applications in soft robotics and actuation systems, where precise control and manipulation are paramount.

### 2.2 Static and Dynamic Performance Parameters

**Figure 2:**
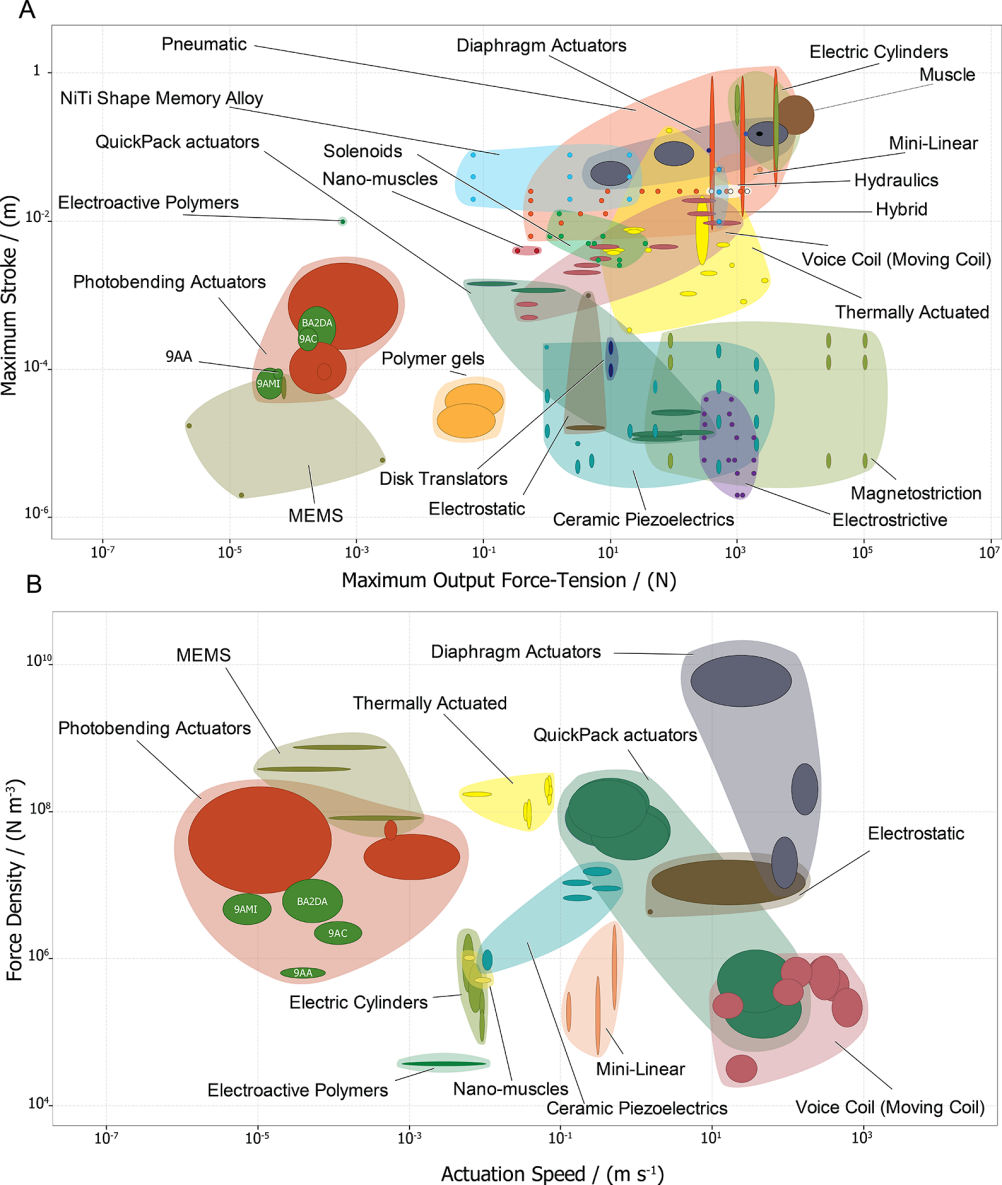
Global materials property plot of the dynamic performance indices for the anthracene-based dynamic crystals compared to other actuator classes. (A) Actuator maximum stroke versus maximum output force and (B) actuator force density versus actuation speed for the four photoresponsive anthracene materials (9AA, 9AC, 9AMI, BA2DA) are coplotted with the same attributes for the main actuator classes. The opaque bubbles represent the range of values of performance indices of a particular material, while the translucent envelopes group materials that belong to the same actuator class.

The maximum actuator force output and maximum free stroke are considered key parameters to describe the performance of an actuator when it is entirely free or restrained (for example, by a sensor or an object that needs to be moved).^8^ The crystals of the four anthracene-based materials were found to have maximum strokes ranging from 1.02·10^–4^ to 7.02·10^–4^ m and a maximum force between 6.75·10^–5^ and 4.63·10^–4^ N, with the values depending on the material (SI Table S1). These key parameters are plotted and compared in Figure 2A with those of the known standard actuator classes as well as the previously reported photobending actuators.^8^ The detailed comparison between these actuators can be found in the Supporting Information (SI Figure S2). Contrary to the anthracene-based crystals and MEMS, electric cylinders and macroactuators such as hydraulic and pneumatic actuators display comparatively larger strokes and output forces. However, considering the maximum stroke, the anthracene-based single crystals suitably occupy the space between hard materials with short strokes and soft materials with larger strokes (Figure 2A). The force output of these crystals is comparable to that of MEMS^51^ and some soft actuators, such as gels and polymer films. Based on the moderate strokes and high force output, the actuating crystals could be suitable for the manipulation or positioning of small objects, as well as for precision and high-resolution tasks. Other possible applications that we envisage for these lightweight organic materials are in dynamic systems that require large deformation rather than high-force outputs, such as optical scanning, laser communications, and biomedical endoscopy.^51,52^

The force density is a useful parameter when choosing a suitable actuator material for some applications with space limitations. Anthracene-based single crystals exhibit significantly higher force densities than electroactive polymers and nanomuscles, which reveals that these crystals can outperform actuators of comparable size (Figure 2B). Considering the cycling performance, the response time is another critical property that determines the maximum actuation frequency. Certain actuators may exhibit a significant disparity in their maximum strokes, spanning across 3 orders of magnitude. This substantial difference between the maximum and minimum stroke renders them unsuitable for comparison in terms of response times. Therefore, rather than response times, here, we consider the actuation *speed* of the unrestrained response to estimate the actuator’s capacity (Figure 2B). The minimum response time can be evaluated based on the maximum stroke and the maximum actuation speed of the actuator. The actuation speeds of the anthracene crystals studied here were determined to be between 1.47·10^–5^ and 2.34·10^–4^ m s^–1^ (SI Τable S1). The fastest response was observed with the 9AA crystals, which have an average response time to reach a maximum displacement of 146 s, and 98 s to reach 50% of the maximum actuation. This material is faster than 9AC, BA2DA, and 9AMI crystals, which respond with response times of 200, 369, and 776 s, respectively.

Since the irradiation conditions can impact the recovery time and the response time of photobendable crystals,^8^ optimal working conditions were established under which the crystals showed the best performance while maintaining their structural integrity and cyclability (SI Table S2). The reported shortest response time is 25 μs for a photoresponsive molecular crystal, which has around 6 min recovery time, may include photothermal effects.^15,53^

Moreover, some diarylethene, azopyridine, and other crystals display photobending with noticeably longer response times, between 35 ms and 5 s, while their recovery times range between 0.5 s and 20 min.^54−57^ Other photobending crystals such as anthracenes and benzoxazole derivatives, require even longer time that ranges between 18 and 44 s, while their recovery times are between 1.2 and 15 min,^20,58^ On the other hand, the so-called photosalient crystals—crystals that are capable of rapid motion or ballistic events under light and respond to light by exploding, jumping, or splitting, probably via a photoinduced phase transition—respond within 5 ms to 33 s, however this can drastically compromise their structural integrity.^32,59^ As shown in Figure 2B, the speed of displacement of the anthracene-based crystals is comparable to MEMS-based devices and thermal actuators as well as to electrically driven piezoelectric and electroactive actuators that are known to respond fast.^52^ The average actuation time required for the crystals to reach their maximum stroke ranged from 2.43 min for 9AA, which was the fastest, to 12.96 min for 9AMI, which was the slowest (SI Table S1).

**Figure 3:**
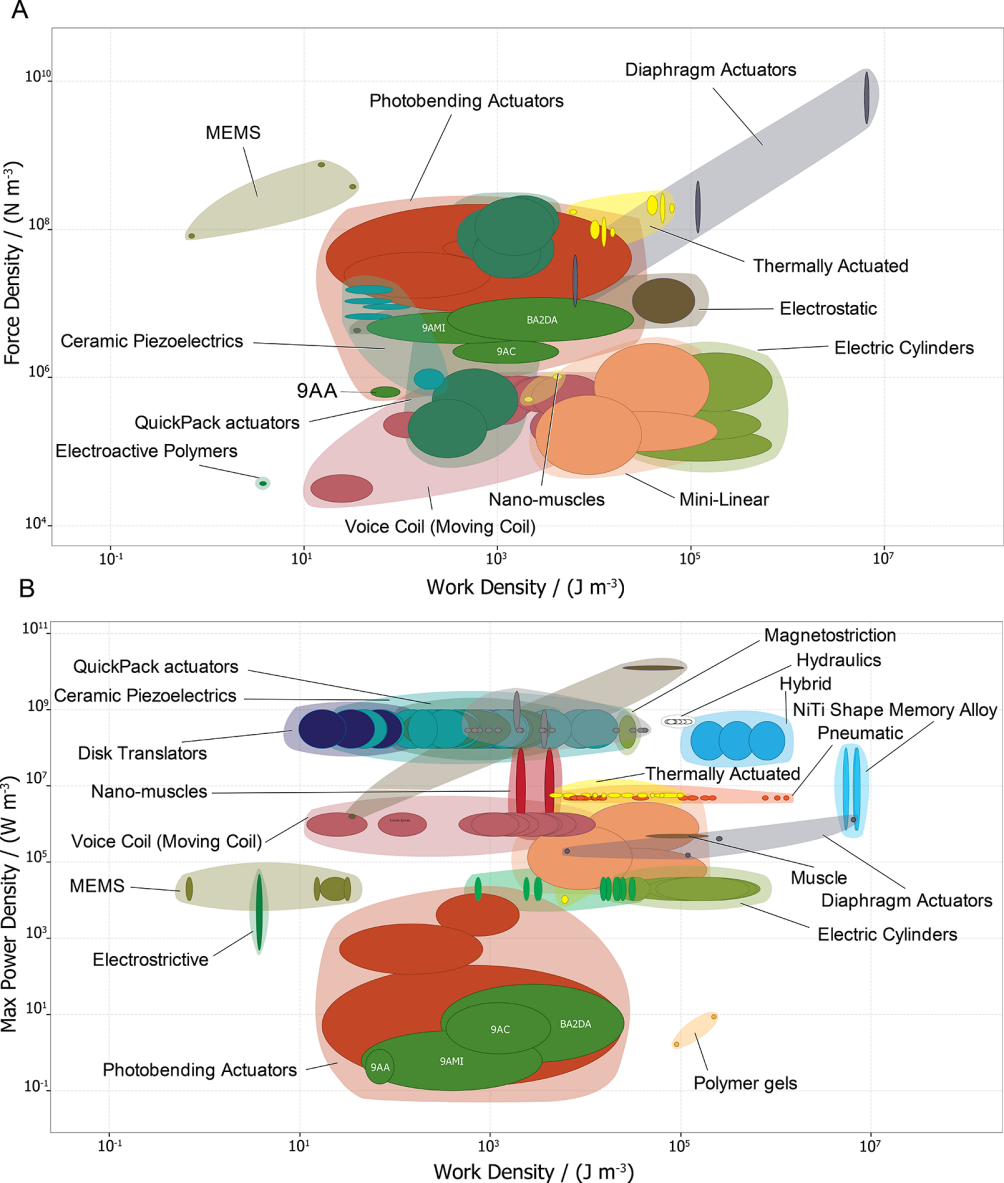
Ashby plots illustrate the relative positioning of anthracene-based crystals within the broader landscape of actuator technologies. (A) Force density versus work density comparison of anthracene-based photomechanical crystals (9AA, 9AC, 9AMI, BA2DA) with various actuator classes, including previously reported photobending actuators.^8^ (B) Maximum power density versus work density, showcasing the dynamic performance of the studied crystals relative to other actuator systems.

The actuators’ dynamic performance is also assessed using indicators like work, power, stroke, and speed.^60^ The maximum work output, *W*_out_, is defined as the energy transferred by the force,
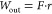
1where *F* is the effective generated force, and *r* is the displacement or stroke.^8^ Crystals of the four anthracene-based materials studied here exhibit maximum work output ranging from 6.91·10^–9^ J to 3.25·10^–7^ J upon bending from their original position (SI Table S1). Their work densities range from 2.51·10^4^ J m^–3^ to 9.62·10^1^ J m^–3^, as depicted in Figure 3A,B. This range is similar to the work density observed in piezoelectric materials. The work density is one of the most essential parameters in the field of materials science and actuator technology, as it provides insight into the amount of work that can be generated per unit volume of the material or device. A high work density indicates that a material can deliver significant mechanical output in a compact space, making it ideal for applications where the space is limited.

### 2.3 Robotic Applications

**Figure 4:**
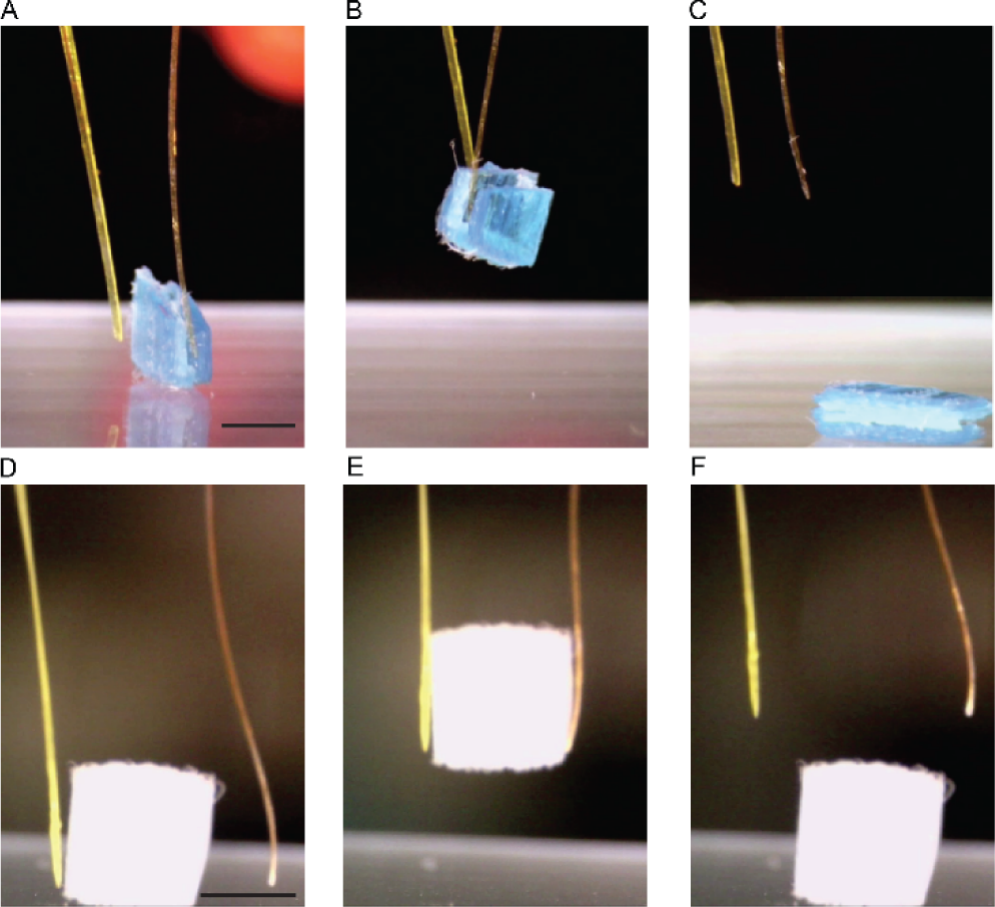
Robotic performance of the crystals of anthracene derivatives. Snapshots are shown of crystals of (A–C) 9AA and BA2DA and (D–F) 9AA and 9AMI, demonstrating contact (A and D), gripping (B and E), and detachment (C and F) of a 3D-printed object upon irradiation with UV light. Scale bar, 500 μm.

In the field of robotics, the actuators play a crucial role in enabling movement and functionality in robotic systems. They are essential components that convert energy into mechanical motion, allowing robots to interact with their environment and perform tasks effectively.^26^ One of the basic robotic applications that require actuators similar to the anthracene-based crystals studied in this research is the development of soft robotic grippers for handling and manipulating delicate objects at a microscopic scale (Figure 4 and SI Figure S3). By incorporating actuators based on dynamic molecular crystals, such as anthracene-based materials, soft robotic grippers can be controlled and actuated by using light from the same source. This innovative approach eliminates the need for traditional motors and mechanical components, offering a more precise and versatile method for manipulating microscopic objects. The use of anthracene-based crystals as actuators in soft robotic grippers opens up new possibilities for applications in fields such as microassembly, biomedicine, and nanotechnology. These crystals respond to light stimuli, allowing for precise and controlled movements that are essential for delicate tasks in confined spaces.^51^ It is worth mentioning that when making robotic applications from these anthracene crystals, it is essential to optimize the setup by selecting crystals that are similar or close in size to ensure that the displacements of both crystals matches; otherwise, the gripper will fail to grasp objects, or the precise manipulation of the objects will be very low. The relationship between the crystal size and the force generated during photomechanical actuation is summarized in SI Figure S5 and SI Table S3, which further emphasizes the need for selecting appropriately sized crystals for effective performance. By harnessing the unique properties of these dynamic molecular crystals, in the future researchers could develop advanced soft robotic systems capable of performing intricate tasks with high precision and efficiency.

## 3 Conclusions

In this study, we analyzed the photomechanical performance of four anthracene-based materials to quantitatively and realistically assess their performance as actuating materials. Crystals of BA2DA and 9AMI were found to be responsive to blue light (470 nm), while crystals of 9AC and 9AA are responsive to UV light (365 nm). The crystals of these materials bent away from the light with the exception of 9AA which bent toward the light. This feature is essential for designing of machines that can perform simple tasks such as gripping in soft robotics. Compared to other photomechanical crystals, anthracene derivatives offer important advantages, including simple synthesis, larger crystal dimensions, and rapid, reversible response, making them one of the most favorable candidates for future applications in microrobotics. Furthermore, the reversible bending allowed us to make customized soft robotic setups such as a microgripper, which was then used to evaluate the application potential of these crystals. We also analyzed the performance indices of the crystals, including their force output, stroke, and actuation speed, showing a comparison with industry-standard actuating materials. The unique properties of these dynamic molecular crystals offer new possibilities for developing advanced soft robotic systems capable of precise and efficient manipulation of delicate objects at a microscopic scale. Future research on this outcome aims to further investigate the underlying reasons of why materials from the same chemical family, such as the anthracene-based derivatives analyzed in this research, exhibit directionally opposing mechanical actuation. This analysis could provide insights into the molecular structures, bonding configurations, and interactions that lead to the observed differences in mechanical responses to light stimuli. Understanding the reasons for the opposing mechanical actuation would require a separate, in-depth analysis of molecular structures, bonding configurations, and interactions, which is a subject of an ongoing research in our laboratories.

## 4 Materials and Methods

### 4.1 Preparation of the Photoresponsive Compounds

The crystals of 9AA, 9AC, 9AMI, and BA2DA were synthesized and/or recrystallized using reported methods.^9,18,48,49^

### 4.2 Force Measurements

To investigate the force generated during the photobending response of the four anthracene derivatives, needle-shaped crystals of 9AC and 9AA were subjected to 365 nm UV illumination from a Ushio SP-11 lamp operating at 30% of its maximum intensity. Meanwhile, the 9AMI and BA2DA crystals were subjected to 470 nm blue light from a Thorlabs M470L3 LED at 100% intensity.

The bending forces exerted by these crystals were quantitatively determined by measuring the deflection of PDMS micropillars^61^ (Supplementary Movies S1–S4). A PDMS micropillar measuring between 250–500 μm in diameter and 1–3 mm in length was prepared to evaluate the performance and force of various crystals. The experimental procedure entailed bending each crystal in contact with the micropillar until minimal displacement of the tip of the pillar was observed. This marked the point at which the force exerted by the crystal on the pillar reached its maximum value and was counteracted by the pillar’s stiffness. By tracking the movement of the displaced pillars, the force applied by the crystal’s tip on each pillar could be calculated using the equation
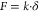
2where *k* represents the stiffness of the micropillar, calculated using the value for the Young’s modulus of PDMS (*E*_PDMS_ = 2.6 MPa),^8,60^ and δ is the displacement of the pillar’s tip. The maximum force output corresponds to the force required to prevent any further displacement of the actuator against the load, known as the “blocking force”.^8^ The illustration of the setup is provided in SI Figure S4.

### 4.3 Analytical Software

The Kinovea software^62,63^ was used to track the displacement of the PDMS micropillar as the crystals pushed against it. Ansys Granta Selector 2020 R2^64^ was used with custom-made script and a database to plot the Ashby plots.

### 4.4 Robotic Application

A tailored robotic (microgripping) experimentation was conducted to assess the potential application of BA2DA and 9AMI crystals (bend away from both UV light and blue LED) and 9AA crystals (bends toward the UV light). The results from these experiments are presented in Figure 4 and SI Movies S5–S7, and they provide conclusive evidence supporting the potential utility of these crystals in specific applications.

## Supporting Information Available

A crystal of 9-anthranaldehyde (9AA) pushing against a PDMS micropillar. The blue light indicates the direction of irradiation with UV light (365 nm). Segments of this movie are shown in SI Figure S1D (MP4)
A crystal of 9-anthracenecarboxylic acid (9AC) pushing against a PDMS micropillar. The blue light indicates the direction of irradiation with UV light (365 nm). Segments of this movie are shown in SI Figure S1A (MP4)
A crystal of (*E*)-2-(9-anthrylmethylene)-1-indanone (9AMI) pushing against a PDMS micropillar. The blue light indicates the direction of irradiation with UV light (365 nm). Segments of this movie are shown in SI Figure S1C (MP4)
A crystal of 1,2-*bis*[(anthracen-9-ylmethylene)amino]ethane (BA2DA) pushing against a PDMS micropillar. The blue light indicates the direction of irradiation with UV light (365 nm). Segments of this movie are shown in SI Figure S1B (MP4)
Demonstration of application of organic crystals in robotics. This series of movies illustrates the application and performance of 9AA and BA2DA crystals utilized as microgrippers. The videos showcase the gripping and detachment processes, highlighting the functional capabilities of these light-activated organic soft microrobots (the recording was sped up by 3 times) (MP4)
Demonstration of application of organic crystals in robotics. This series of movies illustrates the application and performance of 9AA and 9AMI crystals utilized as microgrippers (example 1). The videos showcase the gripping and detachment processes, highlighting the functional capabilities of these light-activated organic soft microrobots (the recording was sped up 3 times) (MP4)
Demonstration of application of organic crystals in robotics. This series of movies illustrates the application and performance of 9AA and 9AMI crystals utilized as microgrippers (example 2). The videos showcase the gripping and detachment processes, highlighting the functional capabilities of these light-activated organic soft microrobots (the recording was sped up 3 times) (MP4)
Descriptions of the Supplementary Movies (PDF)
Figures illustrating the experimental setup, photomechanical actuation, Ashby plots, and line graphs showcasing the performance indices, along with the robotic applications of the crystals. Movies of the crystals’ dynamic bending, gripping, and manipulation under light irradiation. Tables with experimental and analytical data, including photobending performance, force output, and work density across various experimental conditions (PDF)
