# Supplementary material for: Photomechanical Crystals as Light-Activated Organic Soft Microrobots
Source: J Am Chem Soc. 2024 Oct 11;146(44):30174–82. doi: 10.1021/jacs.4c08320 (PMC11544611; doi:10.1021/jacs.4c08320)
Supplement: Supplementary file 8 — ja4c08320_si_008.pdf [file ja4c08320_si_008.pdf]

Description of Additional Supplementary Files

Supplementary Movie 1. A crystal of 9-anthranaldehyde (9AA) pushing against a PDMS micropillar. The blue light indicates the direction of irradiation with UV light (365 nm). Segments of this movie are shown in Supplementary Figure S1D.

Supplementary Movie 2. A crystal of 9-anthracenecarboxylic acid (9AC) pushing against a PDMS micropillar. The blue light indicates the direction of irradiation with UV light (365 nm). Segments of this movie are shown in Supplementary Figure S1A.

Supplementary Movie 3. A crystal of (*E*)-2-(9-anthrylmethylene)-1-indanone (9AMI) pushing against a PDMS micropillar. The blue light indicates the direction of irradiation with UV light (365 nm). Segments of this movie are shown in Supplementary Figure S1C.

Supplementary Movie 4. A crystal of 1,2-bis[(anthracen-9-ylmethylene)amino]ethane (BA2DA) pushing against a PDMS micropillar. The blue light indicates the direction of irradiation with UV light (365 nm). Segments of this movie are shown in Supplementary Figure S1B.

Supplementary Movie 5. Demonstration of application of organic crystals in robotics. This series of movies illustrates the application and performance of 9AA and BA2DA crystals utilized as microgrippers. The videos showcase the gripping and detachment processes, highlighting the functional capabilities of these light-activated organic soft microrobots (the recording was sped up by 3 times).

Supplementary Movie 6. Demonstration of application of organic crystals in robotics. This series of movies illustrates the application and performance of 9AA and 9AMI crystals utilized as microgrippers (example 1). The videos showcase the gripping and detachment processes, highlighting the functional capabilities of these light-activated organic soft microrobots (the recording was sped up 3 times).

Supplementary Movie 7. Demonstration of application of organic crystals in robotics. This series of movies illustrates the application and performance of 9AA and 9AMI crystals utilized as microgrippers (example 2). The videos showcase the gripping and detachment processes, highlighting the functional capabilities of these light-activated organic soft microrobots (the recording was sped up 3 times).
