# Supplementary material for: Photomechanical Crystals as Light-Activated Organic Soft Microrobots
Source: J Am Chem Soc. 2024 Oct 11;146(44):30174–82. doi: 10.1021/jacs.4c08320 (PMC11544611; doi:10.1021/jacs.4c08320)
Supplement: Supplementary file 9 — ja4c08320_si_009.pdf [file ja4c08320_si_009.pdf]

Supporting Information

Photomechanical Crystals as Light-Activated Organic Soft Robots

Ibrahim Tahir,[1,‡] Ejaz Ahmed,[2,‡] Durga Prasad Karothu,[1‡] Filmon Fsehaye,[2,‡] Jad Mahmoud Halabi,[2]
Panče Naumov[1,2,3,4*]

[1]Center for Smart Engineering Materials, New York University Abu Dhabi, PO Box 129188, Abu Dhabi, UAE

[2]Smart Materials Lab, New York University Abu Dhabi, PO Box 129188, Abu Dhabi, UAE

[3]Research Center for Environment and Materials, Macedonian Academy of Sciences and Arts, Bul. Krste Misirkov 2, MK–1000 Skopje, Macedonia

[4]Molecular Design Institute, Department of Chemistry, New York University, 100 Washington Square East, New York, NY 10003, United States

‡ These authors contributed equally.

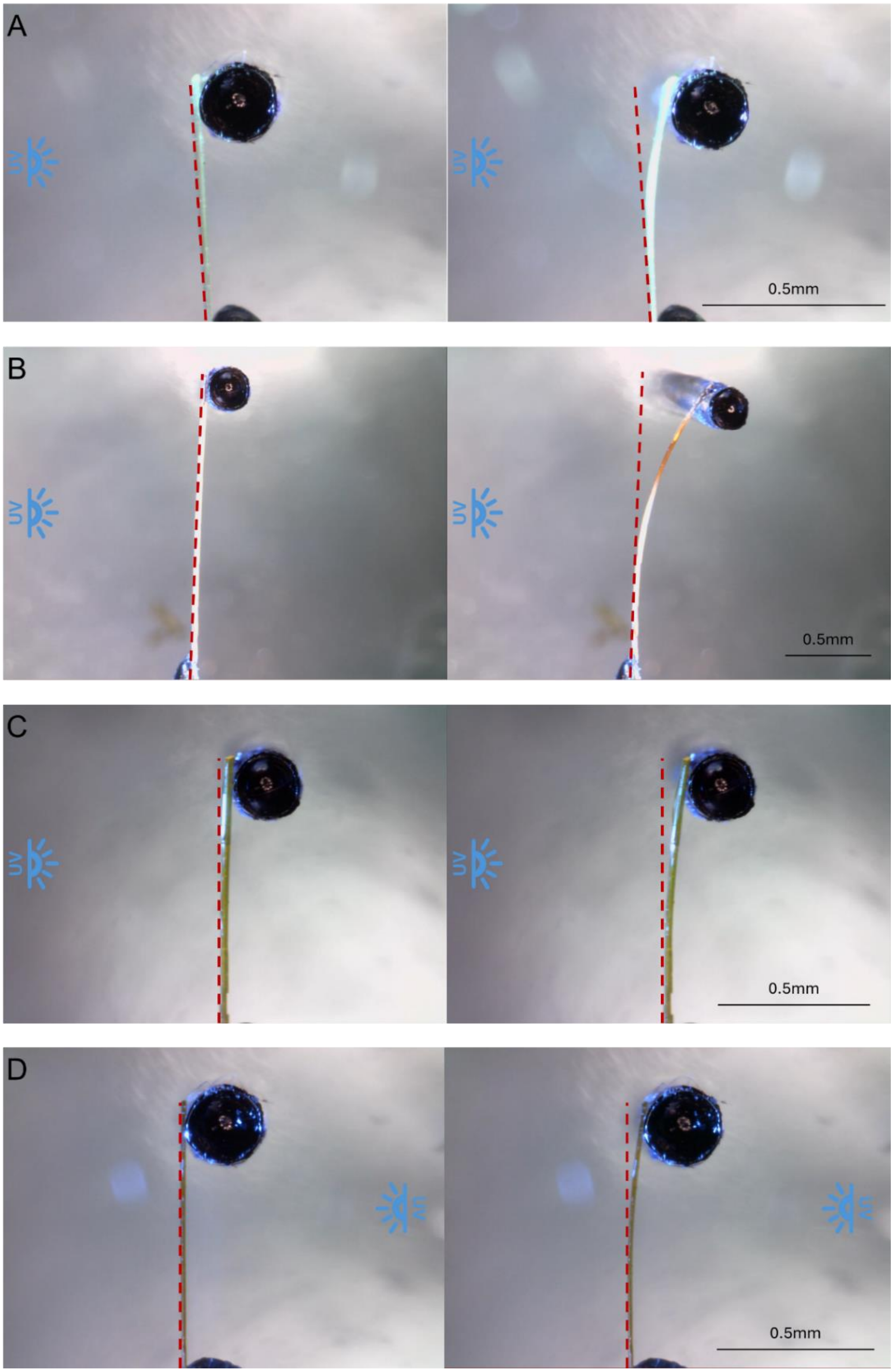

Figure S1. Images of the four compounds as they displace the PDMS micropillars. (A) 9-anthracenecarboxylic acid (9AC). (B) 1,2-bis[(anthracen-9-ylmethylene)amino]ethane (BA2DA). (C) (*E*)-2-(9-anthrylmethylene)-1-indanone (9AMI). (D) 9-anthranaldehyde (9AA). The direction of the UV light is labelled to show the direction of bending relative to the light. The red dashed lines show the original position.

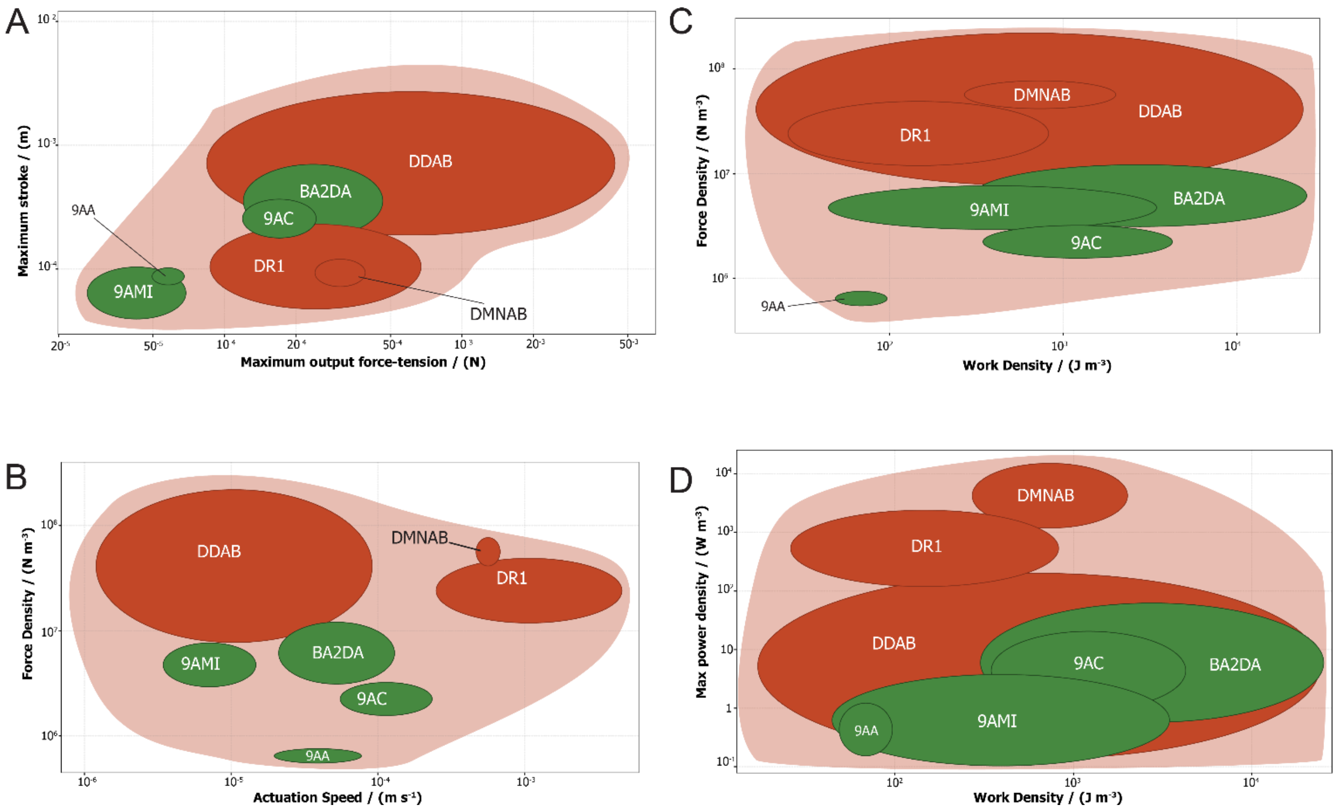

Figure S2. Performance of the anthracene-based single crystals being compared with previously reported azobenzene single crystals. (A) The anthracene crystals have relatively lower stroke and output force. (B) The anthracenes are more clustered at the lower left side of the graph, indicating that they offer better controlled and more stable actuation speeds. (C) The anthracenes overall have lower force density compared to the azobenzenes while offering similar work densities. (D) The power density of the anthracenes is lower than the azobenzenes while they are spread wider in terms of work density.

In comparing the anthracene derivative compounds (9AMI, 9AA, 9AC, and BA2DA, the green set of bubbles) with the azobenzene derivative compounds (DMNAB, DDAB, and DR1, the red set of bubbles) across the Ashby plots shown above, it is evident that while the azobenzenes set generally exhibits superior performance in terms of mechanical properties, the anthracenes offer distinct advantages in applications requiring precision and control. The anthracenes are characterized by moderate values in the maximum output force-tension, force density, and power density, indicating a balanced behavior that prioritizes stability and consistency over peak performance. This makes the anthracenes particularly suitable for specialized or consistent performance in environments where excessive force or rapid response could be detrimental. The anthracenes occupy a more confined region in the plots, suggesting these materials are ideal for controlled and reliable applications, such as in soft robotics, micro-assembly, and biomedical devices, where delicate manipulation and fine-tuned actuation are paramount. While the azobenzene's broader spread across the plots highlights its versatility and adaptability to high-performance environments, the anthracenes outperform in scenarios demanding stable actuation.

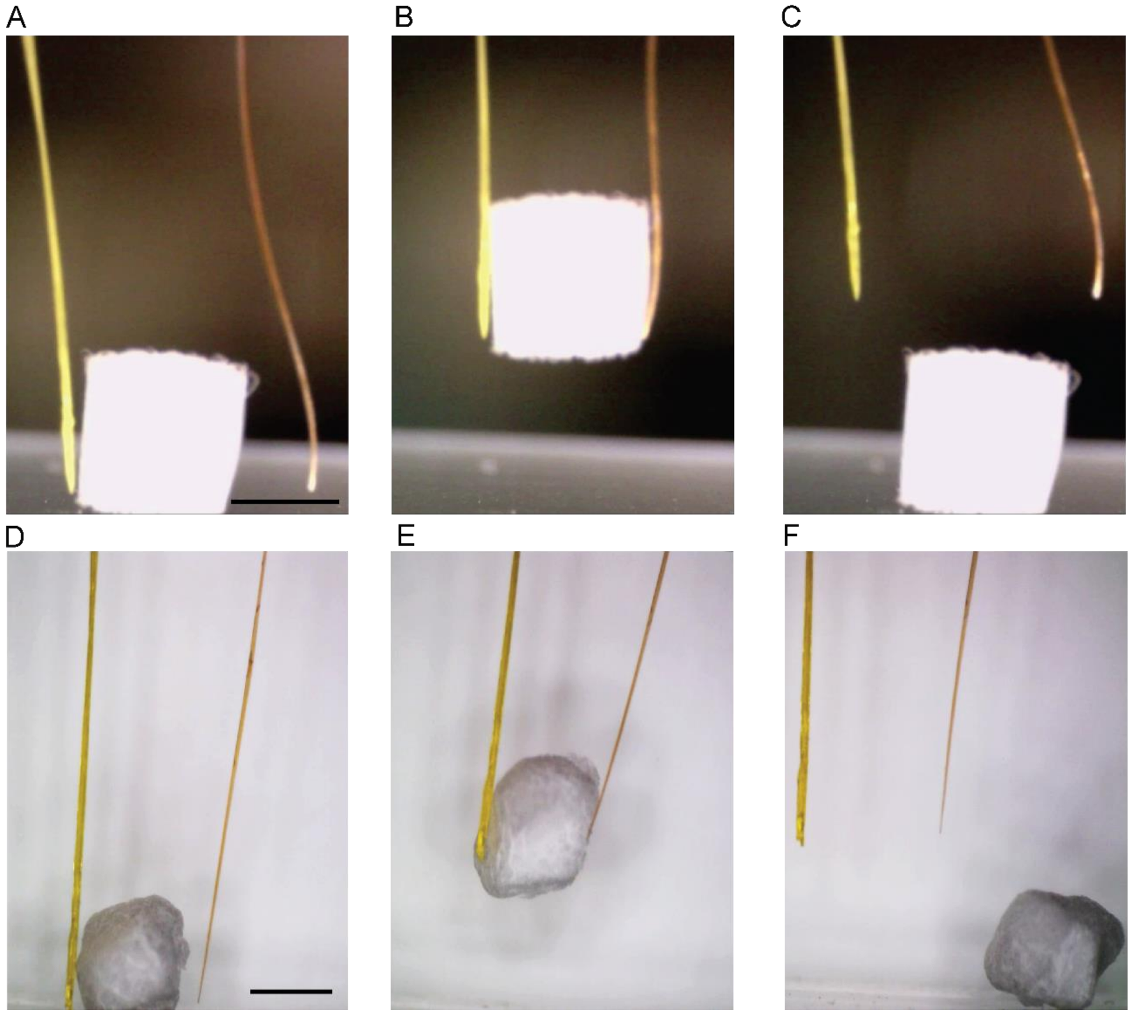

Figure S3. Robotic performance of the crystals of anthracene derivatives. (A–C) Snapshots of 9AA and 9AMI single-crystalline arms demonstrating contact (A) → gripping (B) → (C) detachment of a 3D printed object upon irradiation with UV light. Scale bar, 500 µm. (D–F) Snapshots of 9AA and BA2DA single crystals arms demonstrating contact (D) → gripping (E) → detachment (F) of a microparticle of polystyrene (styrofoam) upon irradiation with UV light. Scale bar, 500 µm.

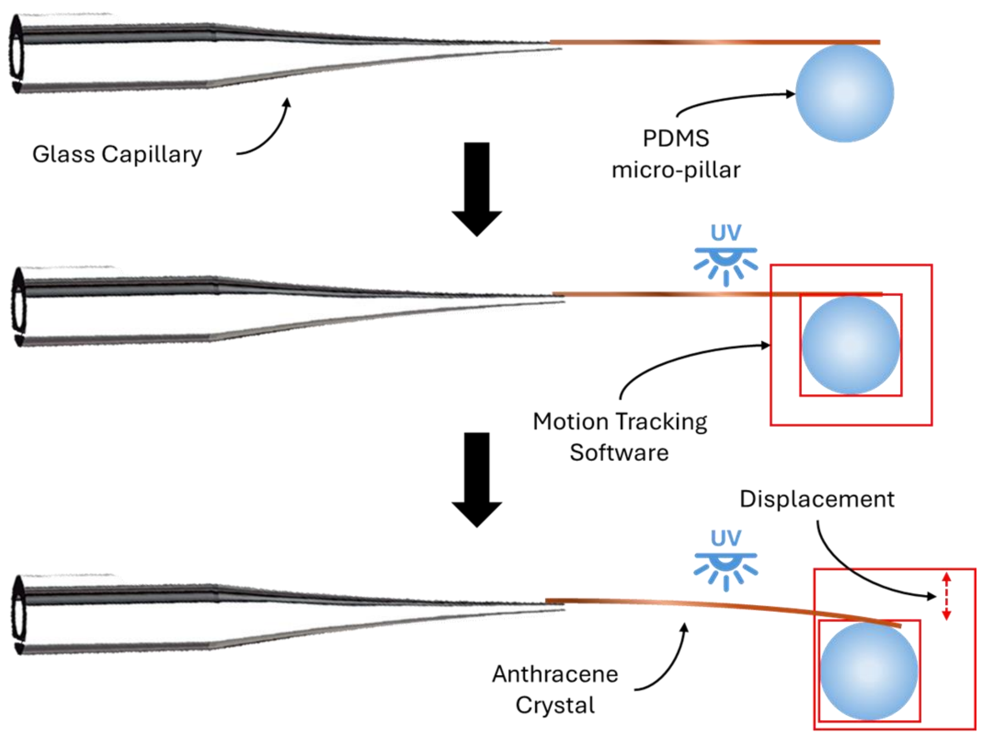

Figure S4. A schematic representation of the experimental setup employed for measuring the force applied by the anthracene crystal on the PDMS micropillars.

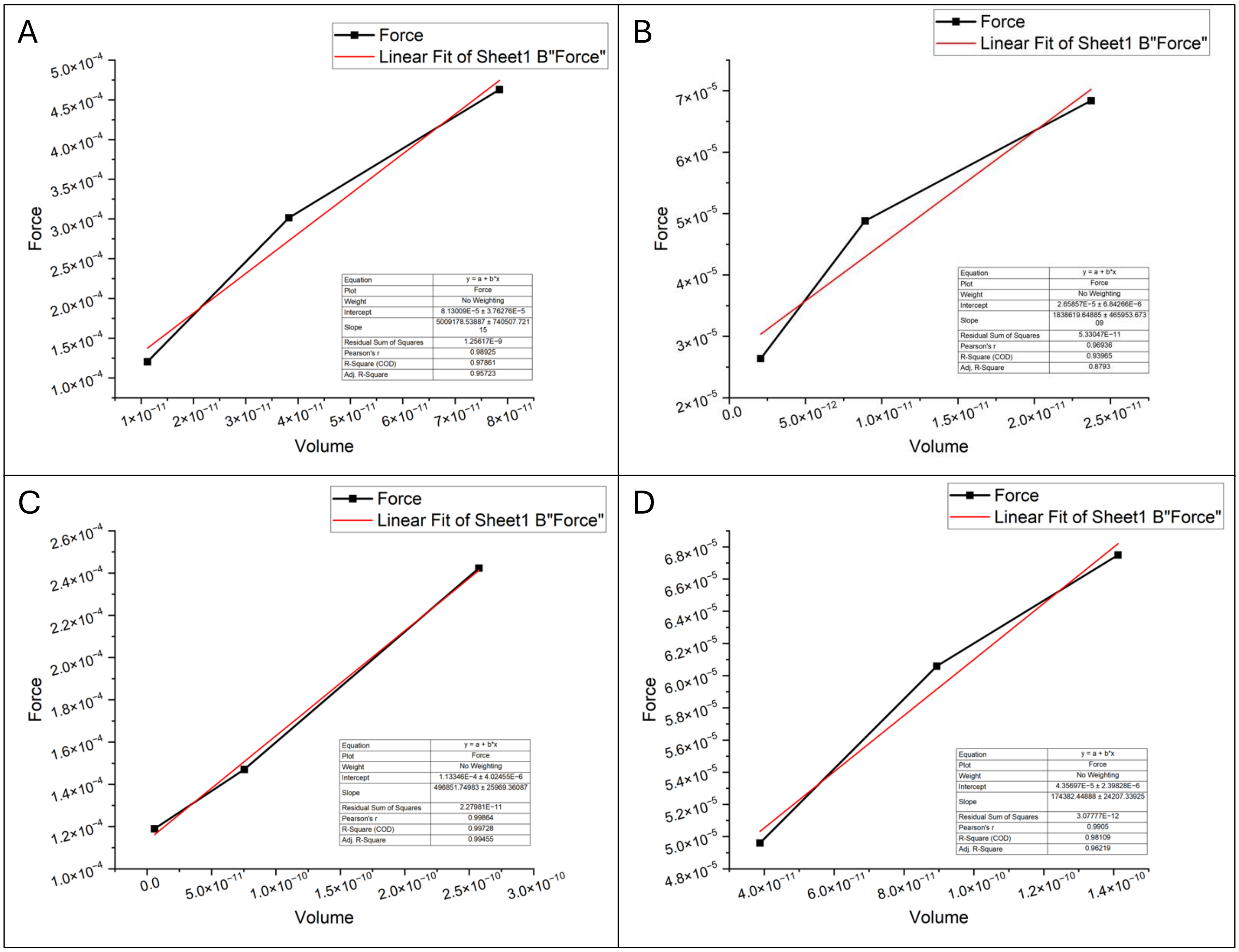

Figure S5. Relationship between crystal volume and force output for four anthracene derivatives: (A) BA2DA, (B) 9AC, (C) 9AMI, and (D) 9AA. Each graph plots the force generated against the volume of the crystal, with a linear fit applied to the data points. The results demonstrate a positive correlation between crystal size and force output.

The relationship between the crystal size and the force generated during photomechanical actuation is well established. The force generated by a crystal is directly related to its volume, as the photodimerization process responsible for bending occurs throughout the irradiated portion of the crystal. However, it is critical to note that there is an optimal size at which the force generated reaches its maximum. This is better understood by examining force density, which accounts for the crystal's volume.

When analyzing the correlation between crystal size and output force, the data suggests a bell-shaped distribution. Crystals that are too small in length tend to exhibit a low aspect ratio between length and thickness, which increases internal stress, thereby reducing their ability to bend effectively. Conversely, crystals that are too large often contain inherent defects, leading to breakage before effective actuation can occur. This trend is clearly illustrated in SI Figure S5, where the force generated by crystals of varying sizes is plotted. The data shows a direct correlation between crystal size and the generated force across all samples. For each compound, the largest and smallest crystals were measured alongside the average force generated by five crystals, with the results summarized in SI Table S3.

Supporting Information Table S1. Performance summary of the photoresponsive anthracene single crystals

Parameters \ Samples	DA2DA	9AMI	9AC-X	9AA
**Max. Displacement (m)**	$7.02 \cdot 10^{-4}$	$1.04 \cdot 10^{-4}$	$3.67 \cdot 10^{-4}$	$1.02 \cdot 10^{-4}$
**Min. Displacement (m)**	$1.82 \cdot 10^{-4}$	$4.00 \cdot 10^{-5}$	$1.80 \cdot 10^{-4}$	$7.52 \cdot 10^{-5}$
**Avg. Displacement (m)**	$4.57 \cdot 10^{-4}$	$7.40 \cdot 10^{-5}$	$2.23 \cdot 10^{-4}$	$9.19 \cdot 10^{-5}$
**Max. Velocity (m s^{-1})**	$1.30 \cdot 10^{-4}$	$1.47 \cdot 10^{-5}$	$2.34 \cdot 10^{-4}$	$7.69 \cdot 10^{-5}$
**Min. Velocity (m s^{-1})**	$2.11 \cdot 10^{-5}$	$3.44 \cdot 10^{-6}$	$5.57 \cdot 10^{-5}$	$1.97 \cdot 10^{-5}$
**Avg. Velocity (m s^{-1})**	$7.92 \cdot 10^{-5}$	$7.01 \cdot 10^{-6}$	$9.34 \cdot 10^{-5}$	$4.79 \cdot 10^{-5}$
**Max. Output Force (N)**	$4.63 \cdot 10^{-4}$	$6.84 \cdot 10^{-5}$	$2.42 \cdot 10^{-4}$	$6.75 \cdot 10^{-5}$
**Min. Output Force (N)**	$1.20 \cdot 10^{-4}$	$2.64 \cdot 10^{-5}$	$1.19 \cdot 10^{-4}$	$4.96 \cdot 10^{-5}$
**Avg. Output Force (N)**	$3.02 \cdot 10^{-4}$	$4.88 \cdot 10^{-5}$	$1.47 \cdot 10^{-4}$	$6.06 \cdot 10^{-5}$
**Max. Power Output (W)**	$1.00 \cdot 10^{-9}$	$1.29 \cdot 10^{-11}$	$4.64 \cdot 10^{-10}$	$8.65 \cdot 10^{-11}$
**Min. Power Output (W)**	$4.24 \cdot 10^{-11}$	$2.29 \cdot 10^{-12}$	$1.18 \cdot 10^{-10}$	$2.21 \cdot 10^{-11}$
**Avg. Power Output (W)**	$4.71 \cdot 10^{-10}$	$5.92 \cdot 10^{-12}$	$2.14 \cdot 10^{-10}$	$4.80 \cdot 10^{-11}$
**Max. Power Density (W m^{-3})**	$6.15 \cdot 10^{1}$	$3.75 \cdot 10^{0}$	$2.04 \cdot 10^{1}$	$1.21 \cdot 10^{0}$
**Min. Power Density (W m^{-3})**	$5.82 \cdot 10^{-1}$	$1.06 \cdot 10^{-1}$	$9.56 \cdot 10^{-1}$	$1.56 \cdot 10^{-1}$
**Avg. Power Density (W m^{-3})**	$2.95 \cdot 10^{1}$	$1.37 \cdot 10^{0}$	$8.24 \cdot 10^{-8}$	$6.87 \cdot 10^{-1}$
**Max. Work Output (J)**	$3.25 \cdot 10^{-7}$	$7.09 \cdot 10^{-9}$	$8.90 \cdot 10^{-8}$	$6.91 \cdot 10^{-9}$
**Min. Work Output (J)**	$2.19 \cdot 10^{-8}$	$1.06 \cdot 10^{-9}$	$2.15 \cdot 10^{-8}$	$3.73 \cdot 10^{-9}$
**Avg. Work Output (J)**	$1.66 \cdot 10^{-7}$	$3.92 \cdot 10^{-9}$	$3.62 \cdot 10^{-8}$	$5.65 \cdot 10^{-9}$
**Max. Work Output Density (J m^{-3})**	$2.51 \cdot 10^{4}$	$3.44 \cdot 10^{3}$	$4.24 \cdot 10^{3}$	$9.62 \cdot 10^{1}$
**Min. Work Output Density (J m^{-3})**	$3.02 \cdot 10^{2}$	$4.45 \cdot 10^{1}$	$3.46 \cdot 10^{2}$	$4.87 \cdot 10^{1}$
**Avg. Work Output Density (J m^{-3})**	$1.05 \cdot 10^{4}$	$1.19 \cdot 10^{3}$	$1.33 \cdot 10^{3}$	$6.99 \cdot 10^{1}$

Supporting Information Table S2. Data collected to calculate the photobending performance of the photoresponsive anthracene single crystals. The table shows data obtained from 20 single crystals in total (5 crystals of each compound)

BA2DA					
**Sample number**	1	2	3	4	5
**Actuation time (min)**	$4.18 \cdot 10^{0}$	$8.63 \cdot 10^{0}$	$5.41 \cdot 10^{0}$	$7.9 \cdot 10^{0}$	$5.48 \cdot 10^{0}$
**Displacement (m)**	$3.9 \cdot 10^{-4}$	$1.82 \cdot 10^{-4}$	$7.2 \cdot 10^{-4}$	$6.86 \cdot 10^{-4}$	$4.7 \cdot 10^{-4}$
**Output force (N)**	$2.4 \cdot 10^{-4}$	$1.20 \cdot 10^{-4}$	$4.63 \cdot 10^{-4}$	$4.52 \cdot 10^{-4}$	$2.69 \cdot 10^{-4}$
**Volume of the crystal (m^3)**	$1.12 \cdot 10^{-11}$	$7.28 \cdot 10^{-11}$	$1.63 \cdot 10^{-11}$	$1.24 \cdot 10^{-11}$	$7.85 \cdot 10^{-11}$
**Force Density (N m^{-3})**	$1.81 \cdot 10^{7}$	$1.65 \cdot 10^{6}$	$2.84 \cdot 10^{7}$	$3.65 \cdot 10^{7}$	$3.42 \cdot 10^{6}$
**Work Output (J)**	$6.28 \cdot 10^{-8}$	$2.19 \cdot 10^{-8}$	$3.25 \cdot 10^{-7}$	$3.10 \cdot 10^{-7}$	$1.9 \cdot 10^{-7}$
**Work Output Density (J m^{-3})**	$5.60 \cdot 10^{3}$	$3.2 \cdot 10^{2}$	$1.99 \cdot 10^{4}$	$2.51 \cdot 10^{4}$	$1.39 \cdot 10^{3}$
**Power Output (J s^{-1})**	$2.50 \cdot 10^{-}10$	$4.24 \cdot 10^{-11}$	$1.00 \cdot 10^{-9}$	$7.30 \cdot 10^{-}10$	$3.33 \cdot 10^{-10}$
**Power Density (W m^{-1})**	$2.23 \cdot 10^{1}$	$5.82 \cdot 10^{-1}$	$6.15 \cdot 10^{1}$	$5.90 \cdot 10^{1}$	$4.24 \cdot 10^{0}$
**9AMI**					
**Actuation Time (min)**	$5.46 \cdot 10^{0}$	$1.77 \cdot 10^{1}$	$6.96 \cdot 10^{0}$	$1.53 \cdot 10^{1}$	$1.94 \cdot 10^{1}$
**Displacement (m)**	$8.2 \cdot 10^{-5}$	$6.7 \cdot 10^{-5}$	$4.00 \cdot 10^{-5}$	$1.4 \cdot 10^{-4}$	$8.53 \cdot 10^{-5}$
**Output force (N)**	$5.29 \cdot 10^{-5}$	$4.1 \cdot 10^{-5}$	$2.64 \cdot 10^{-5}$	$6.84 \cdot 10^{-5}$	$5.63 \cdot 10^{-5}$
**Volume of the crystal (m^3)**	$1.21 \cdot 10^{-11}$	$2.40 \cdot 10^{-12}$	$2.37 \cdot 10^{-11}$	$2.6 \cdot 10^{-12}$	$4.30 \cdot 10^{-12}$
**Force Density (N m^{-3})**	$4.39 \cdot 10^{6}$	$1.67 \cdot 10^{7}$	$1.11 \cdot 10^{6}$	$3.32 \cdot 10^{7}$	$1.31 \cdot 10^{7}$
**Work Output (J)**	$4.24 \cdot 10^{-9}$	$2.43 \cdot 10^{-9}$	$1.6 \cdot 10^{-9}$	$7.9 \cdot 10^{-9}$	$4.80 \cdot 10^{-9}$

**Work Output Density (J m^{-3})**	$3.52 \cdot 10^{2}$	$1.1 \cdot 10^{3}$	$4.45 \cdot 10^{1}$	$3.44 \cdot 10^{3}$	$1.12 \cdot 10^{3}$
**Power Output (J s^{-1})**	$1.29 \cdot 10^{-11}$	$2.29 \cdot 10^{-12}$	$2.53 \cdot 10^{-12}$	$7.73 \cdot 10^{-12}$	$4.13 \cdot 10^{-12}$
**Power Density (W m^{-1})**	$1.7 \cdot 10^{0}$	$9.55 \cdot 10^{-1}$	$1.6 \cdot 10^{-1}$	$3.75 \cdot 10^{0}$	$9.60 \cdot 10^{-1}$
**9AC^{-}X**					
**Actuation Time (min)**	$7.70 \cdot 10^{-1}$	$3.15 \cdot 10^{0}$	$3.28 \cdot 10^{0}$	$6.3 \cdot 10^{0}$	$3.46 \cdot 10^{0}$
**Displacement (m)**	$1.80 \cdot 10^{-4}$	$1.84 \cdot 10^{-4}$	$1.90 \cdot 10^{-4}$	$3.67 \cdot 10^{-4}$	$1.93 \cdot 10^{-4}$
**Output force (N)**	$1.19 \cdot 10^{-4}$	$1.22 \cdot 10^{-4}$	$1.25 \cdot 10^{-4}$	$2.42 \cdot 10^{-4}$	$1.27 \cdot 10^{-4}$
**Volume of the crystal (m^{3})**	$3.95 \cdot 10^{-11}$	$2.3 \cdot 10^{-11}$	$5.38 \cdot 10^{-11}$	$2.58 \cdot 10^{-10}$	$5.78 \cdot 10^{-12}$
**Force Density (N m^{-3})**	$3.1 \cdot 10^{6}$	$5.99 \cdot 10^{6}$	$2.32 \cdot 10^{6}$	$9.41 \cdot 10^{5}$	$2.20 \cdot 10^{7}$
**Work Output (J)**	$2.15 \cdot 10^{-8}$	$2.24 \cdot 10^{-8}$	$2.37 \cdot 10^{-8}$	$8.90 \cdot 10^{-8}$	$2.45 \cdot 10^{-8}$
**Work Output Density (J m^{3-})**	$5.43 \cdot 10^{2}$	$1.10 \cdot 10^{3}$	$4.41 \cdot 10^{2}$	$3.46 \cdot 10^{2}$	$4.24 \cdot 10^{3}$
**Power Output (J s^{-1})**	$4.64 \cdot 10^{-10}$	$1.18 \cdot 10^{-10}$	$1.21 \cdot 10^{-10}$	$2.46 \cdot 10^{-10}$	$1.18 \cdot 10^{-10}$
**Power Density (W m^{-1})**	$1.18 \cdot 10^{1}$	$5.84 \cdot 10^{0}$	$2.24 \cdot 10^{0}$	$9.56 \cdot 10^{-1}$	$2.4 \cdot 10^{1}$
**9AA**					
**Actuation Time (min)**	$2.61 \cdot 10^{0}$	$1.33 \cdot 10^{0}$	$1.33 \cdot 10^{0}$	$5.19 \cdot 10^{0}$	$1.67 \cdot 10^{0}$
**Displacement (m)**	$9.74 \cdot 10^{-5}$	$7.52 \cdot 10^{-5}$	$1.2 \cdot 10^{-4}$	$1.2 \cdot 10^{-4}$	$8.23 \cdot 10^{-5}$
**Output force (N)**	$6.42 \cdot 10^{-5}$	$4.96 \cdot 10^{-5}$	$6.75 \cdot 10^{-5}$	$6.73 \cdot 10^{-5}$	$5.43 \cdot 10^{-5}$
**Volume of the crystal (m^{3})**	$1.19 \cdot 10^{-10}$	$3.88 \cdot 10^{-11}$	$7.99 \cdot 10^{-11}$	$1.41 \cdot 10^{-10}$	$6.84 \cdot 10^{-11}$
**Force Density (N m^{-3})**	$5.41 \cdot 10^{5}$	$1.28 \cdot 10^{6}$	$8.45 \cdot 10^{5}$	$4.77 \cdot 10^{5}$	$7.93 \cdot 10^{5}$
**Work Output (J)**	$6.25 \cdot 10^{-9}$	$3.73 \cdot 10^{-9}$	$6.91 \cdot 10^{-9}$	$6.88 \cdot 10^{-9}$	$4.47 \cdot 10^{-9}$
**Work Output Density (J m^{-3})**	$5.27 \cdot 10^{1}$	$9.62 \cdot 10^{1}$	$8.65 \cdot 10^{1}$	$4.87 \cdot 10^{1}$	$6.53 \cdot 10^{1}$
**Power Output (J s^{-1})**	$3.99 \cdot 10^{-11}$	$4.67 \cdot 10^{-11}$	$8.65 \cdot 10^{-11}$	$2.21 \cdot 10^{-11}$	$4.46 \cdot 10^{-11}$
**Power Density (W m^{-1})**	$3.37 \cdot 10^{-1}$	$1.21 \cdot 10^{0}$	$1.8 \cdot 10^{0}$	$1.56 \cdot 10^{-1}$	$6.52 \cdot 10^{-1}$

Supporting Information Table S3. Summary of the data highlighting the relationship between crystal volume and the generated force, demonstrating the variability in force output across different crystal sizes.

**BA2DA**			
**Max Volume (m^{3})**	$7.85 \cdot 10^{-11}$	**Max Force Output (N)**	$4.63 \cdot 10^{-04}$
**Average Volume (m^{3})**	$3.82 \cdot 10^{-11}$	**Average Force Output (N)**	$3.02 \cdot 10^{-04}$
**Min Volume (m^{3})**	$1.12 \cdot 10^{-11}$	**Min Force Output (N)**	$1.20 \cdot 10^{-04}$
**9AC**			
**Max Volume (m^{3})**	$2.58 \cdot 10^{-10}$	**Max Force Output (N)**	$2.42 \cdot 10^{-04}$
**Average Volume (m^{3})**	$7.54 \cdot 10^{-11}$	**Average Force Output (N)**	$1.47 \cdot 10^{-04}$
**Min Volume (m^{3})**	$5.78 \cdot 10^{-12}$	**Min Force Output (N)**	$1.19 \cdot 10^{-04}$
**9AMI**			
**Max Volume (m^{3})**	$2.37 \cdot 10^{-11}$	**Max Force Output (N)**	$6.84 \cdot 10^{-05}$
**Average Volume (m^{3})**	$8.91 \cdot 10^{-12}$	**Average Force Output (N)**	$4.88 \cdot 10^{-05}$
**Min Volume (m^{3})**	$2.06 \cdot 10^{-12}$	**Min Force Output (N)**	$2.64 \cdot 10^{-05}$
**9AA**			
**Max Volume (m^{3})**	$1.41 \cdot 10^{-10}$	**Max Force Output (N)**	$6.75 \cdot 10^{-05}$
**Average Volume (m^{3})**	$8.94 \cdot 10^{-11}$	**Average Force Output (N)**	$6.06 \cdot 10^{-05}$
**Min Volume (m^{3})**	$3.88 \cdot 10^{-11}$	**Min Force Output (N)**	$4.96 \cdot 10^{-05}$
